# Supplementary material for: Maternal thyroid hormone receptor β activation in mice sparks brown fat thermogenesis in the offspring
Source: Nat Commun. 2023 Oct 24;14:6742. doi: 10.1038/s41467-023-42425-w (PMC10597992; doi:10.1038/s41467-023-42425-w)
Supplement: Supplementary file 1 — Supplementary Information [file 41467_2023_42425_MOESM1_ESM.docx]

**Supplementary Information for**

**Maternal thyroid hormone receptor β activation sparks brown fat thermogenesis in the offspring**

Rebecca Oelkrug, Lisbeth Harder, Mehdi Pedaran, Anne Hoffmann, Beke Kolms, Julica Inderhees, Sogol Gachkar, Julia Resch, Kornelia Johann, Olaf Jöhren, Kerstin Krause, and Jens Mittag


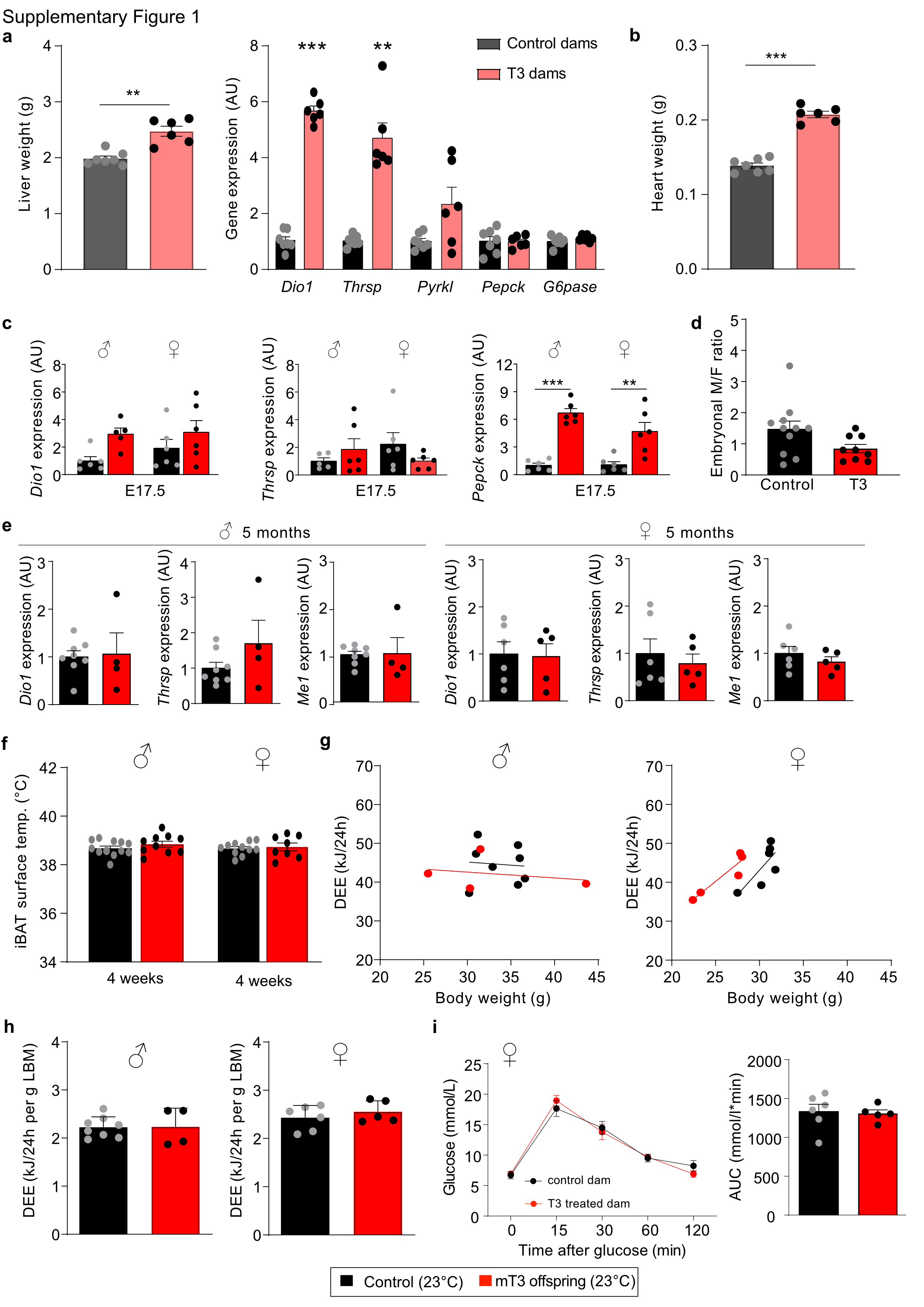


**Figure S1:** a) Liver weight and hepatic gene expression in pregnant females treated with T3 (light red, n=6) or control (gray, n=7). b) Heart weight of these dams. c) Hepatic gene expression in the embryos of these dams at embryonic day E17.5 (n=5-6). d) Male to female ratio in the embryos of T3-treated (n=9) and control (n=11) mothers. e) Hepatic gene expression of male and female offspring of mothers treated with T3 during pregnancy (red, n=4 males or n=5 females) or controls (black, n=8 males and n=6 females) at 5 months of age. f) Surface temperature of iBAT in these offspring after weaning at 4 weeks of age (n=8-12). g) ANCOVA analysis of daily energy expenditure (DEE) per gram body weight of male and female mT3 offspring. h) Daily energy expenditure (DEE) per gram lean body mass (LBM, n=4-8). i) GTT i.p. in female offspring of mothers treated with T3 during pregnancy (red, n=5) or controls (black, n=6) and area under the curve for the response. All values are mean ± SEM. **: p<0.01; ***:p<0.001 for maternal treatment. *Dio1: Deiodinase type I, Me1: malic enzyme, Thrsp: thyroid hormone responsive hepatic gene, Pyrkl: Pyruvate kinase liver, Pepck: Phosphoenolpyruvate carboxykinase; G6Pase: Glucose-6-phosphatase*. Biological replicates were obtained from 3 - 4 litters per group. Statistical details are provided in Suppl Data 1.


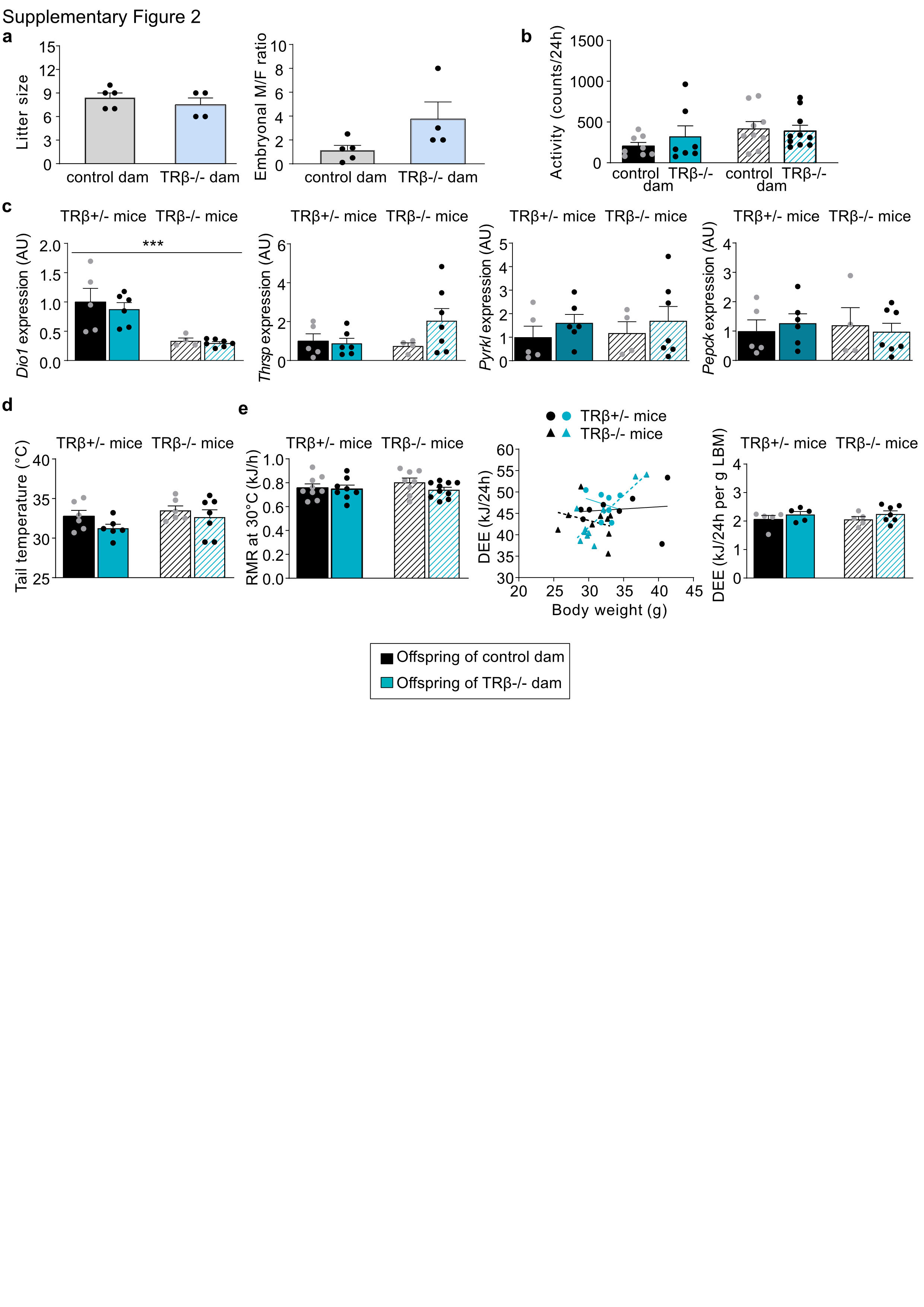


**Figure S2:** a) Litter size and embryonal male to female ratio in TRβ+/- (n=5) and TRβ-/- (n=4) dams. b) Locomotor activity in male offspring of TRβ-/- mothers (blue, n=7-10) or control dams (black, n=8-9) with TRβ+/- (left columns, solid color) or TRβ-/- genotype (right columns, striped). c) Hepatic gene expression in these offspring. d) Tail temperature in these offspring (Control offspring: n = 6 per group, TRβ-/- offspring: n = 6 from TRβ+/- dam and n = 7 from TRβ-/- dam). e) Resting metabolic rate (RMR) at 30°C and daily energy expenditure with body weight as covariate and normalized on body lean mass in the offspring (Control offspring: n = 5-8 from TRβ+/- dam and n = 5-7 from TRβ-/- dam, TRβ-/- offspring: n = 5-9 from TRβ+/- dam and n = 7-10 from TRβ-/- dam). All values are mean ± SEM. ***:p<0.001 for genotype effect. *Dio1: Deiodinase type I, Thrsp: thyroid hormone responsive hepatic gene, Pyrkl: Pyruvate kinase liver, Pepck: Phosphoenolpyruvate carboxykinase; G6pase: Glucose-6-phosphatase*. Biological replicates were obtained from 3 - 4 litters per group. Statistical details are provided in Suppl Data 1.


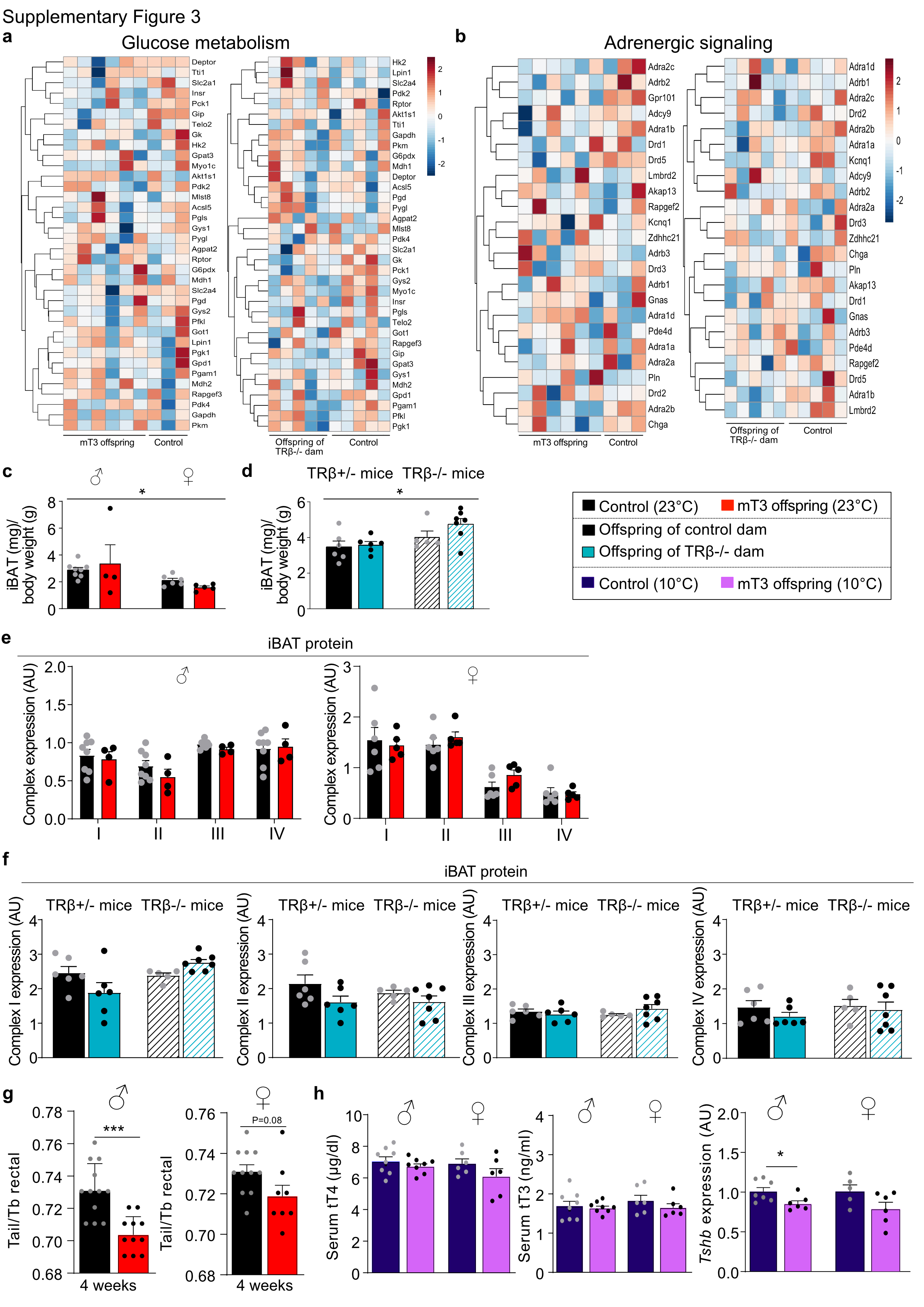


**Figure S3**: a) Heatmap for glucose metabolism genes (GO: 0006006) in iBAT of male offspring of mothers treated with T3 during pregnancy or controls (left) based on microarray gene expression data. Heatmap for glucose metabolism genes in iBAT of male offspring of TRβ-/- mothers or control dams (right). b) Heatmap for the groups in A) for genes involved in adrenergic signaling (GO: 0071875). c) iBAT weight in male and female offspring of mothers treated with T3 during pregnancy (red, n=4 males and n=5 females) or controls (black, n=8 males and n=6 females). d) iBAT weight of male offspring of TRβ-/- mothers (blue, n=6-7) or control dams (black, n=5-6) with TRβ+/- (left columns, solid color) or TRβ-/- genotype (right columns, striped). e) iBAT protein levels in the animals of c). f) iBAT protein levels of the animals in d). g) Tail temperature normalized to rectal temperature in 4 week old male and female offspring of mothers treated with T3 during pregnancy (red, n=11 males and n=8 females) or controls (black, n=12 males and n=11 females). h) Serum total T4, total T3 and pituitary *Tshb* mRNA expression in male offspring of mothers treated with T3 during pregnancy (purple, n=6-8 males and n=6 females) or controls (darkblue, n=7-8 males and n=5-6 females) that were exposed to 10°C for 4 weeks. All values are mean ± SEM with individual values in circles. *: p<0.05 and ***: p<0.001 for maternal T3 treatment. Biological replicates were obtained from 3 - 4 litters per group (mT3 offspring (23°C) and TRβ offspring) and 4 – 6 litters per group (mT3 offspring (10°C)). Statistical details are provided in Suppl Data 1.

**Supplementary Table 1**

List of genes that were regulated in opposite directions when comparing offspring of control mothers to T3-treated mothers and control mothers to mothers lacking TRβ using microarray gene expression data. Expression values are given as log2 as provided by the microarray. Differential gene expression analysis was conducted utilizing the Linear Models for Microarray Data (LIMMA) approach, which is implemented in the R package limma. This method seamlessly integrates linear modeling and empirical Bayes techniques, offering a robust framework for the accurate identification of differentially expressed genes. Only genes with an expression of log2>5 and a fold change of |FC|>1.25 were included. p-values are uncorrected. After correction for multiple testing, only *Olfr237-ps1* remains significant. n=4-6 per group. Biological replicates were obtained from 3 - 4 litter per group.

| Gene Symbol | Control offspring average  (log2) | T3 offspring average  (log2) | Fold-change | p-value uncorr. | Control offspring average  (log2) | TRβ-/- offspring average  (log2) | Fold-change | p-value uncorr. |
| --- | --- | --- | --- | --- | --- | --- | --- | --- |
| *Chek2* | 7.58 | 7.14 | -1.35 | 0.009 | 7.01 | 7.34 | 1.25 | 0.020 |
| *Mppe1* | 9.47 | 9.11 | -1.29 | 0.034 | 9.17 | 9.52 | 1.27 | 0.046 |
| *Lysmd3* | 8.49 | 8.12 | -1.30 | 0.004 | 8.26 | 8.63 | 1.29 | 0.025 |
| *Usp11* | 6.46 | 5.85 | -1.53 | 0.004 | 6.05 | 6.45 | 1.32 | 0.025 |
| *Myo5b* | 6.17 | 5.67 | -1.41 | 0.005 | 5.91 | 6.40 | 1.41 | 0.049 |
| *Pfkfb4* | 6.58 | 6.01 | -1.49 | 0.007 | 5.94 | 6.51 | 1.48 | 0.031 |
| *Rbm4* | 6.36 | 5.94 | -1.34 | 0.043 | 6.08 | 6.69 | 1.52 | 0.012 |
| *Nab2* | 6.64 | 6.15 | -1.41 | 0.006 | 6.11 | 6.73 | 1.53 | 0.036 |
|  |  |  |  |  |  |  |  |  |
| *Usp26* | 6.40 | 6.87 | 1.38 | 0.008 | 6.80 | 6.11 | -1.61 | 0.017 |
| *Hmox1* | 8.40 | 9.05 | 1.57 | 0.005 | 9.05 | 8.36 | -1.61 | 0.010 |
| *H2-Q1* | 6.18 | 6.93 | 1.69 | 0.003 | 6.61 | 5.97 | -1.56 | 0.015 |
| *Itgb2* | 5.21 | 5.71 | 1.42 | 0.018 | 5.68 | 5.09 | -1.50 | 0.037 |
| *Chst1* | 5.91 | 6.24 | 1.26 | 0.034 | 6.16 | 5.58 | -1.49 | 0.020 |
| *Gbgt1* | 5.56 | 6.06 | 1.41 | 0.050 | 5.57 | 5.03 | -1.45 | 0.021 |
| *Il2rg* | 6.82 | 7.29 | 1.39 | 0.024 | 7.41 | 6.89 | -1.43 | 0.029 |
| *Vcam1* | 6.82 | 7.33 | 1.42 | 0.025 | 7.06 | 6.54 | -1.43 | 0.033 |
| *Man1c1* | 7.25 | 7.60 | 1.28 | 0.004 | 7.57 | 7.08 | -1.41 | 0.009 |
| *Gm5535* | 5.18 | 5.99 | 1.75 | 0.001 | 5.80 | 5.34 | -1.38 | 0.025 |
| *Olfr237-ps1* | 6.55 | 7.07 | 1.43 | 0.000 | 7.01 | 6.57 | -1.36 | 0.001 |
