# Supplementary material for: Maternal thyroid hormone receptor β activation in mice sparks brown fat thermogenesis in the offspring
Source: Nat Commun. 2023 Oct 24;14:6742. doi: 10.1038/s41467-023-42425-w (PMC10597992; doi:10.1038/s41467-023-42425-w)
Supplement: Supplementary file 3 — Description of Additional Supplementary Files [file 41467_2023_42425_MOESM3_ESM.docx]

**Description of additional supplementary files for**

**Maternal thyroid hormone receptor β activation sparks brown fat thermogenesis in the offspring**

Rebecca Oelkrug, Lisbeth Harder, Mehdi Pedaran, Anne Hoffmann, Beke Kolms, Julica Inderhees, Sogol Gachkar, Julia Resch, Kornelia Johann, Olaf Jöhren, Kerstin Krause, and Jens Mittag

**Supplementary Data 1**

Statistical details of performed analysis. *: p<0.05; **: p<0.01; ***: p<0.001; all statistical tests were two-sided (when relevant).

**Supplementary Data 2**

List of metabolites that were detected in serum of T3-treated and control dams. A two-tailed student´s t-test with false discovery rate (FDR) correction was used to detect significant differences between the groups and differentially expressed metabolites were marked in yellow with bold text. n = 6 per group.
