# Supplementary material for: Maternal thyroid hormone receptor β activation in mice sparks brown fat thermogenesis in the offspring
Source: Nat Commun. 2023 Oct 24;14:6742. doi: 10.1038/s41467-023-42425-w (PMC10597992; doi:10.1038/s41467-023-42425-w)
Supplement: Supplementary file 6 — Reporting Summary [file 41467_2023_42425_MOESM6_ESM.pdf]

nature portfolio

Corresponding author(s): JENS MITTAG

Last updated by author(s): Sep 7, 2023

Reporting Summary

Nature Portfolio wishes to improve the reproducibility of the work that we publish. This form provides structure for consistency and transparency in reporting. For further information on Nature Portfolio policies, see our Editorial Policies and the Editorial Policy Checklist.

Statistics

For all statistical analyses, confirm that the following items are present in the figure legend, table legend, main text, or Methods section.

n/a	Confirmed	
[ ]	[x]	The exact sample size (*n*) for each experimental group/condition, given as a discrete number and unit of measurement
[ ]	[x]	A statement on whether measurements were taken from distinct samples or whether the same sample was measured repeatedly
[ ]	[x]	The statistical test(s) used AND whether they are one- or two-sided<br>*Only common tests should be described solely by name; describe more complex techniques in the Methods section.*
[x]	[ ]	A description of all covariates tested
[ ]	[x]	A description of any assumptions or corrections, such as tests of normality and adjustment for multiple comparisons
[ ]	[x]	A full description of the statistical parameters including central tendency (e.g. means) or other basic estimates (e.g. regression coefficient) AND variation (e.g. standard deviation) or associated estimates of uncertainty (e.g. confidence intervals)
[ ]	[x]	For null hypothesis testing, the test statistic (e.g. *F*, *t*, *r*) with confidence intervals, effect sizes, degrees of freedom and *P* value noted<br>*Give P values as exact values whenever suitable.*
[x]	[ ]	For Bayesian analysis, information on the choice of priors and Markov chain Monte Carlo settings
[x]	[ ]	For hierarchical and complex designs, identification of the appropriate level for tests and full reporting of outcomes
[x]	[ ]	Estimates of effect sizes (e.g. Cohen's *d*, Pearson's *r*), indicating how they were calculated

Our web collection on statistics for biologists contains articles on many of the points above.

Software and code

Policy information about availability of computer code

Data collection	TSE PhenoMaster software (V5.8.1, V6.2.0 and V6.5.3, TSE Systems, Germany), CaloBox software (PhenoSys GmbH, Germany)
Data analysis	Excel 2016/2010/365 Version 2023, GraphPad Prism Version 7-9, oligo R package (v1.50.0), Biobase (v2.46), R Version 4.1.0 with package arrayQualityMetrics (v3.42) and limma (v3.42), Compound Discoverer 3.1 (ThermoFisher Scientific, Germany) with MetaboAnalyst 5.0

For manuscripts utilizing custom algorithms or software that are central to the research but not yet described in published literature, software must be made available to editors and reviewers. We strongly encourage code deposition in a community repository (e.g. GitHub). See the Nature Portfolio guidelines for submitting code & software for further information.

Data

Policy information about availability of data

All manuscripts must include a data availability statement. This statement should provide the following information, where applicable:

- Accession codes, unique identifiers, or web links for publicly available datasets
- A description of any restrictions on data availability
- For clinical datasets or third party data, please ensure that the statement adheres to our policy

Source data are provided with this paper or have been deposited in the ArrayExpress database at EMBL-EBI (www.ebi.ac.uk/arrayexpress) under accession number E-MTAB-12587 for the microarray study, and at MassIVE (https://massive.ucsd.edu/ProteoSAFe/static/massive.jsp) under accession number MSV000091042 for the metabolomics data respectively.

Research involving human participants, their data, or biological material

Policy information about studies with human participants or human data. See also policy information about sex, gender (identity/presentation), and sexual orientation and race, ethnicity and racism.

Reporting on sex and gender	N/A
Reporting on race, ethnicity, or other socially relevant groupings	N/A
Population characteristics	N/A
Recruitment	N/A
Ethics oversight	N/A

Note that full information on the approval of the study protocol must also be provided in the manuscript.

Field-specific reporting

Please select the one below that is the best fit for your research. If you are not sure, read the appropriate sections before making your selection.

[x] Life sciences [ ] Behavioural & social sciences [ ] Ecological, evolutionary & environmental sciences

For a reference copy of the document with all sections, see nature.com/documents/nr-reporting-summary-flat.pdf

Life sciences study design

All studies must disclose on these points even when the disclosure is negative.

Sample size	Sample size for animal study was calculated with an a priori power analysis, which in Germany is required by law for ethical approval.
Data exclusions	An outlier test was performed routinely on all datasets prior analysis using GraphPad Prism with preestablished criteria (ROUT method, Q=1%). If any data were identified as outliers and removed, they have been marked as such in the source data file.
Replication	Experiments were carried out in independent animals (ie. biological replicates) and the number of replicates is given by individual data points in each figure. Outlier tests were conducted to identify non-successful replicates, thus all presented data points constitute successful individual replicates.
Randomization	For experiments with wildtypes vs genetically modified mice, no randomization was possible, the mice were sorted into the groups based on sex and genotype. For experiments only conducted with wildtypes, the females were randomly assigned to either control or T3 treatment.
Blinding	The animals were recorded with continuous ID numbers, which do not provide information on the genotype.

Reporting for specific materials, systems and methods

We require information from authors about some types of materials, experimental systems and methods used in many studies. Here, indicate whether each material, system or method listed is relevant to your study. If you are not sure if a list item applies to your research, read the appropriate section before selecting a response.

Materials & experimental systems

n/a	Involved in the study	
[ ]	[x]	Antibodies
[x]	[ ]	Eukaryotic cell lines
[x]	[ ]	Palaeontology and archaeology
[ ]	[x]	Animals and other organisms
[x]	[ ]	Clinical data
[x]	[ ]	Dual use research of concern
[x]	[ ]	Plants

Methods

n/a	Involved in the study	
[x]	[ ]	ChIP-seq
[x]	[ ]	Flow cytometry
[x]	[ ]	MRI-based neuroimaging

Antibodies

Antibodies used	rabbit anti-UCP1 antibody (customized rabbit antibody raised against UCP1, 1:1000, previously used in (63))<br>mouse anti-total OxPhos antibody cocktail (#45-8099, 1:1000, Invitrogen Germany)

	goat anti-rabbit-IgG, #P0448, DAKO, Denmark, 1:1000<br>goat anti-mouse-IgG, #P0447, DAKO, Denmark, 1:1000
Validation	The UCP1 antibody has been validated using UCP1 KO animals, and did not show any residual stainings at the appropriate size in the KO animals. The secondary antibodies are a well established standard and have been used in several publications by us and others. They have been validated for off target effects in our lab by omitting the respective primary antibody.

Animals and other research organisms

Policy information about studies involving animals; ARRIVE guidelines recommended for reporting animal research, and Sex and Gender in Research

Laboratory animals	Mice were kept on a constant 12-hour light/12-hour dark cycle at 23 ± 1°C and 40-60% humidity with free access to food and water (breeding diet #1314 from Altromin, Germany, 14 % fat, 27 % protein, 59 % carbohydrates, metabolized energy: ~3.339 kcal/g or 13.97 kJ/g). All animals were on a C57BL/6NCrl background strain, which was controlled by genetic typing regularly. The respective ages of the animals are provided in the results or figure legends.
Wild animals	No wild animals were used in this study.
Reporting on sex	With the exception of the data in Fig 3, male and female mice were used and the data are provided separated by sex.
Field-collected samples	No field-collected samples were used in this study.
Ethics oversight	Animals were monitored daily, euthanized using carbondioxide or isoflurane in combination with cervical dislocation, and procedures were approved by the Ministerium für Energiewende, Klimaschutz, Umwelt und Natur MEKUN Schleswig-Holstein, Germany.

Note that full information on the approval of the study protocol must also be provided in the manuscript.
